# Supplementary material for: Cucurbitacin B-, E-, and I-Induced Browning of White Adipocytes Is Promoted by the Inhibition of Phospholipase D2
Source: Int J Mol Sci. 2022 Dec 6;23(23):15362. doi: 10.3390/ijms232315362 (PMC9740502; doi:10.3390/ijms232315362)
Supplement: Supplementary file 1 [file ijms-23-15362-s001.zip › ijms-2025889-supplementary.pdf]

Table S1. Chemical and physical properties of Cucurbitacin B, E and I.

	Cucurbitacin B	Cucurbitacin E	Cucurbitacin I
Molecular Weight	558.7	556.7	514.6
Molecular Formula	$C_{32}H_{46}O_8$	$C_{32}H_{44}O_8$	$C_{30}H_{42}O_7$
XLogP3-AA	2.6	3.2	2.7
Hydrogen Bond Donor Count	3	3	4
Hydrogen Bond Acceptor Count	8	8	7
Rotatable Bond Count	6	6	4
Exact Mass	558.31926842	556.30361836	514.29305367
Monoisotopic Mass	558.31926842	556.30361836	514.29305367
Topological Polar Surface Area	138	138	132
Heavy Atom Count	40	40	37
Formal Charge	0	0	0
Complexity	1210	1270	1160
Isotope Atom Count	0	0	0
Defined Atom Stereocenter Count	9	8	8
Undefined Atom Stereocenter Count	0	0	0
Defined Bond Stereocenter Count	1	1	1
Undefined Bond Stereocenter Count	0	0	0
Covalently-Bonded Unit Count	1	1	1
Compound Is Canonicalized	Yes	Yes	Yes
